# Effects of stent-assisted coiling in comparison with flow diversion on intracranial aneurysms

**DOI:** 10.3389/fneur.2022.937536

**Published:** 2022-11-08

**Authors:** Hao Guo, Jian-Feng Liu, Cong-Hui Li, Ji-Wei Wang, Hui Li, Bu-Lang Gao

**Affiliations:** Department of Neurosurgery, The First Hospital, Hebei Medical University, Shijiazhuang, China

**Keywords:** wide-neck, intracranial aneurysms, stent-assisted embolization, flow diversion, effect

## Abstract

**Objective:**

The aim of this study was to investigate the efficacy and complications of stent-assisted coiling in comparison with flow diversion for wide-necked intracranial aneurysms.

**Methods:**

Patients with wide-necked intracranial aneurysms who were treated with stent-assisted coiling or flow diversion were respectively, enrolled into the stent-assisted coiling or flow diversion treatment group. The clinical and angiographic data were analyzed.

**Results:**

A total of 61 patients with intracranial aneurysms underwent stent-assisted coiling, including 35 (57.4%) female and 26 (42.6%) male patients with 21 (34.4%) ruptured and 40 (65.6%) unruptured aneurysms. Also, 53 patients underwent deployment of flow-diverting devices, including 30 (56.6%) female and 23 (43.4%) male patients with 25 (47.2%) ruptured and 28 (52.8%) unruptured aneurysms. Stent-assisted coiling was performed successfully in 60 patients with 63 stents deployed, and immediate aneurysm occlusion was complete occlusion in 38 (62.3%) aneurysms, residual neck in 12 (19.7%), and residual aneurysm in 10 (16.4%). Procedure-related complications included in-stent thrombosis in three (4.9%) patients, coil protrusion in three (4.9%), and re-rupture of one (1.6%) aneurysm, with a total complication rate of 11.5%. In the flow diversion group, a pipeline embolization device alone was deployed in each of the 24 (45.3%) patients, adjunctive coiling combined with a pipeline device in 29 (54.7%), and double pipeline devices in each of the 6 (11.3%) patients. Immediately after treatment, complete occlusion was achieved in 3 (5.7%) patients with adjunctive coiling, residual neck in 3 (5.7%), and residual aneurysm in 47 (88.7%). Procedure-related complications included aneurysm rebleeding in one patient (1.9%). Clinical and angiographic follow-up was performed 13–49 months (median 29) after the procedure for 49 (80.3%) patients with stent-assisted coiling, with complete aneurysm occlusion in 27 (55.1%) aneurysms, residual neck in 3 (6.1%), residual aneurysm in 5 (10.2%), and recurrence in 14 (28.6%). Follow-up was performed for 14–37 (median 25) months in 45 (84.9%) patients with flow diversion treatment, with complete occlusion in 39 (86.7%) patients, residual neck in 5 (11.1%), residual aneurysm in 1 (2.2%), and no aneurysm recurrence.

**Conclusions:**

Stent-assisted coiling comes with more complications but fewer permanent aneurysm occlusions than flow diverters, and flow diverters are superior to stent-assisted coiling in the treatment of wide-necked intracranial aneurysms, especially in the long-term effect.

## Introduction

The publication of the international subarachnoid aneurysm trial of neurosurgical treatment vs. endovascular coiling in 2,143 patients with ruptured intracranial aneurysms has established the role of endovascular embolization in treating cerebral aneurysms, and since then, endovascular embolization has been applied as a routine for cerebral aneurysms, especially ruptured aneurysms (1). However, endovascular management of wide-necked cerebral aneurysms remains a technical challenge because of the risk of coil protrusion, possibly leading to thrombosis and parent artery compromise. Several endovascular techniques have been applied for wide-necked aneurysms, including balloon- or stent-assisted coiling, flow diversion, and the WEB aneurysm embolization system (Sequent Medical, Aliso Viejo, CA, USA) (2–5). A stent can have an endurable support for coils within the aneurysm sac and prevent coils from escaping out of the sac. With use of flow diverters or stents, complication rates may be higher than those with selective coil embolization or balloon-assisted coiling due to thrombogenicity of the devices and a need for dual-antiplatelet administration. Use of antiplatelet therapy in stent-assisted coiling or flow diversion in acute subarachnoid hemorrhage may cause high rates of early adverse events, elevated thromboembolic complications, increased risks of intracranial hemorrhage and rebleeding from a ruptured aneurysm, increased morbidity and mortality, and potential of infarction secondary to vasospasm (2, 6–8). Stent-assisted coiling was initially developed to overcome the limitations of coiling alone such as aneurysmal neck remnant and coil protrusion into the artery (9). However, technical challenges remain with the stent-assisted coiling technique, including difficulty navigating the coiling microcatheter through the interstices of the stent, stent malposition, and incomplete coiling besides long-term recurrence of aneurysms. The advent of flow-diverting devices has facilitated the treatment of cerebral aneurysms. However, few studies have been performed to directly compare the safety and efficiency of stent-assisted coiling with flow diversion for the treatment of wide-necked ruptured and unruptured intracranial aneurysms. It was thus hypothesized that both stent-assisted coiling and flow diversion could be safely and efficiently applied to treat ruptured and unruptured cerebral aneurysms. This study was consequently performed to investigate the safety and effect of stent-assisted coiling and flow diversion in the treatment of wide-necked intracranial aneurysms.

## Materials and methods

This retrospective one-center study was approved by the ethics committee of our hospital, and all patients or their family members provided signed informed consent to participate. Patients who underwent stent-assisted coiling or deployment of flow-diverting devices using the pipeline embolization device (PED, Medtronic, Irvine, CA, USA) for wide-necked intracranial aneurysms between January 2016 and June 2020 were enrolled into two groups, namely, stent-assisted coiling and flow diversion treatment. Wide-necked aneurysms were referred to those with a neck diameter of ≥ 4 mm or a dome-to-neck ratio of <2. The inclusion criteria were consecutive patients with wide-necked ruptured or unruptured aneurysms confirmed by computed tomography angiography (CTA) or digital subtraction angiography, who were treated with stent-assisted coiling or flow diversion, and who were without contraindiction to the endovascular treatment or contrast agent. The exclusion criteria were patients with subarachnoid hemorrhage caused by other non-aneurysmal diseases or trauma, ruptured cerebral aneurysms treated without use of stents, with contraindications for use of contrast agents, and with severe heart, renal, and liver diseases. In this study, wide-necked intracranial aneurysms were treated either with stent-assisted coiling or flow diversion, and assignment of the patients into these two groups was based on the desire and selection of the patients after informed consent. In the initial period of this study, stent-assisted coiling was performed more frequently while, in the later period, more patients with wide-necked cerebral aneurysms experienced deployment of flow diverters with a better understanding of the advantages of these flow diverters.

In patients with unruptured aneurysms, thromboelastography was performed 3 days before the embolization procedure to test the response of antiplatelet medications, and the dosage of dual antiplatelet medications was adjusted according to the test outcome to maintain the inhibition rate of arachidonic acid more than 50%, the inhibitive rate of adenosine diphosphate over 30%, and the maximal amplitude of adenosine diphosphate curve at 31–47 mm.

Stent-assisted coiling and flow diversion treatment of cerebral aneurysms were performed by neurosurgeons with 5–10 years of experience in endovascular treatment, with the patient in supine position under general anesthesia. In patients with ruptured aneurysms, aspirin (300 mg) and clopidogrel (300 mg) were administered *via* nasogastric feeding 3 h before the embolization procedure, and in patients with unruptured aneurysms, aspirin 100 mg/day and clopidogrel 75 mg/day were administered 5 days before the procedure. After puncture of one common femoral artery and insertion of an arterial sheath and a guiding catheter, cerebral angiography was performed. In patients with deployment of a PED device alone, an appropriate PED device was selected and deployed to cover the aneurysm neck, with the PED device long enough to anchor at both the proximal and distal sides of the aneurysm neck. For aneurysms treated with stent-assisted coiling or PED plus adjunctive coiling, an appropriate stent or PED device was selected according to the size of the parent artery and aneurysm and sent to the vessel distal to the aneurysm. After deployment of coils within the aneurysm sac, the stent or PED device was navigated to the aneurysm and deployed partially or completely. After stent deployment, 2,000 IU heparin was slowly injected intravenously for systemic heparinization. If the procedure exceeded 2 h, 1,000 IU heparin was injected intravenously once every hour. Immediately or within 24 h after embolization, all patients underwent head CT scan and repeated head CT scan was performed within 72 h for monitoring possible subarachnoid hemorrhage and hydrocephalus. Anticoagulation was continued with low molecular heparin 4,000 IU injected subcutaneously twice daily for 2 days and continued afterward with aspirin 100 mg administered orally once per day for 3 months. Clopidogrel at the dose of 75 mg was administered orally once daily for 1 month.

Periprocedural complications, stents used, occlusion status, rebleeding of aneurysms, thrombosis, coil escape, and clinical outcomes were recorded. Angiographic follow-up was performed once half a year, 1, 3, and 5 years following embolization. Aneurysm occlusion was evaluated with the Raymond–Roy grade, with complete occlusion as grade I, residual neck as grade II, and residual aneurysm as grade III (10). Aneurysm recanalization was diagnosed if opacification of the aneurysm was seen to increase in amount.

### Statistical analysis

The SPSS 19.0 software (IBM, Chicago, IL, USA) was used for statistical analysis. Measurement data were expressed as mean ± standard deviation and tested with the *t*-test if in normal distribution. If not in normal distribution, the measurement data were presented as median (range) and tested with the Wilcoxon rank-sum test. Enumeration data were presented as numbers and percentages and tested with the chi-square test or Fisher's exact probability method. *p* < 0.05 was set as the statistically significant level.

## Results

### Patient's data

Among 61 patients undergoing stent-assisted coiling, there were 35 (57.4%) female and 26 (42.6%) male patients, with an age range of 34–76 (mean ± 15) years, including 21 (34.4%) with ruptured and 40 (65.6%) with unruptured aneurysms (Table 1). The most frequent location of aneurysm was internal carotid artery (ICA) (*n* = 36 or 59.0%), especially the posterior communicating artery (Pcom) segment (*n* = 22, 36.1%), followed by intracranial vertebral artery (*n* = 9 or 14.8%). Among 21 patients with ruptured aneurysms, the Hunt–Hess grade was I in 5 (23.8%) patients, II in 13 (61.9%), III in 1 (4.8%), and IV in 1 (4.8%). Most aneurysms were between 3 and 10 mm (*n* = 40, 65.6%), 14 (23.0%) aneurysms were between 10 and 25 mm, with 6 (9.8%) aneurysm ≤ 3 mm and 1 (1.6%) aneurysm > 25 mm.

**Table 1:** Demography and treated aneurysms.

**Variables**		**Stent-assisted coiling (61)**	**Flow diversion (53)**	**P**
F/M		35/26	30/23	0.56
Age (y)		34–76 (mean 56 ± 15)	29–80 (mean 55 ± 12)	0.54
Unruptured/ruptured		40/21	28/25	0.32
ICA	Pcom	22 (36.1%)	16 (30.2%)	0.87
	Cavernous segment	4 (6.6%)	7 (13.2%)	
	OOP segment	8 (13.1%)	4 (7.5%)	
	ICA bifurcation	2 (3.3%)	0	
	ACA A1 segment	1 (1.6%)	2 (3.8%)	
	Acom	6 (9.8%)	5 (9.4%)	
	MCA M1 segment	3 (4.9%)	8 (15.1%)	
	Intracranial VA	9 (14.8%)	6 (11.3%)	
	BA	6 (9.8%)	5 (9.4%)	
Aneurysm size	≤ 3 mm	6 (9.8%)	4 (7.5%)	0.87
	3 mm <D ≤ 10 mm	40 (65.6%)	30 (56.6%)	
	10 mm <D ≤ 25 mm	14 (23.0%)	18 (34.0%)	
	D > 25 mm	1 (1.6%)	1 (1.9%)	
Hunt-Hess grade	I	5 (23.8%)	9 (36%)	0.87
	II	13 (61.9%)	11 (44%)	
	III	1 (4.8%)	5 (20%)	
	IV	1 (4.8%)	0	

ICA, internal carotid artery; ACA, anterior cerebral artery; MCA, middle cerebral artery; OOP, ophthalmic or Paraclinoid; Pcom, posterior communicating artery; Acom, anterior communicating artery; VA, vertebral artery; BA, basilar artery.

Among 53 patients undergoing deployment of flow-diverting devices, there were 30 (56.6%) female and 23 (43.4%) male patients with an age range of 29–80 (mean 55 ± 12) years, including 25 (47.2%) patients with ruptured and 28 (52.8%) with unruptured aneurysms. The most frequent location of aneurysm was ICA (*n* = 27 or 50.9%), especially the Pcom segment (*n* = 16 or 30.2%), followed by the middle cerebral artery (*n* = 8 or 15.1%). Among 25 patients with ruptured aneurysms, the Hunt–Hess grade was I in 9 (36%) patients, II in 11 (44%), and III in 5 (20%). Most aneurysms (*n* = 30, 56.6%) were between 3 and 10 mm, and 18 (34.0%) were between 10 and 25 mm, with 4 (7.5%) aneurysms ≤ 3 mm and 1 (1.9%) aneurysm > 25 mm.

No significant (*p* > 0.05) difference existed in the age, sex, aneurysm size and location, and the Hunt–Hess grade.

### Endovascular treatment

**Figure 1:**
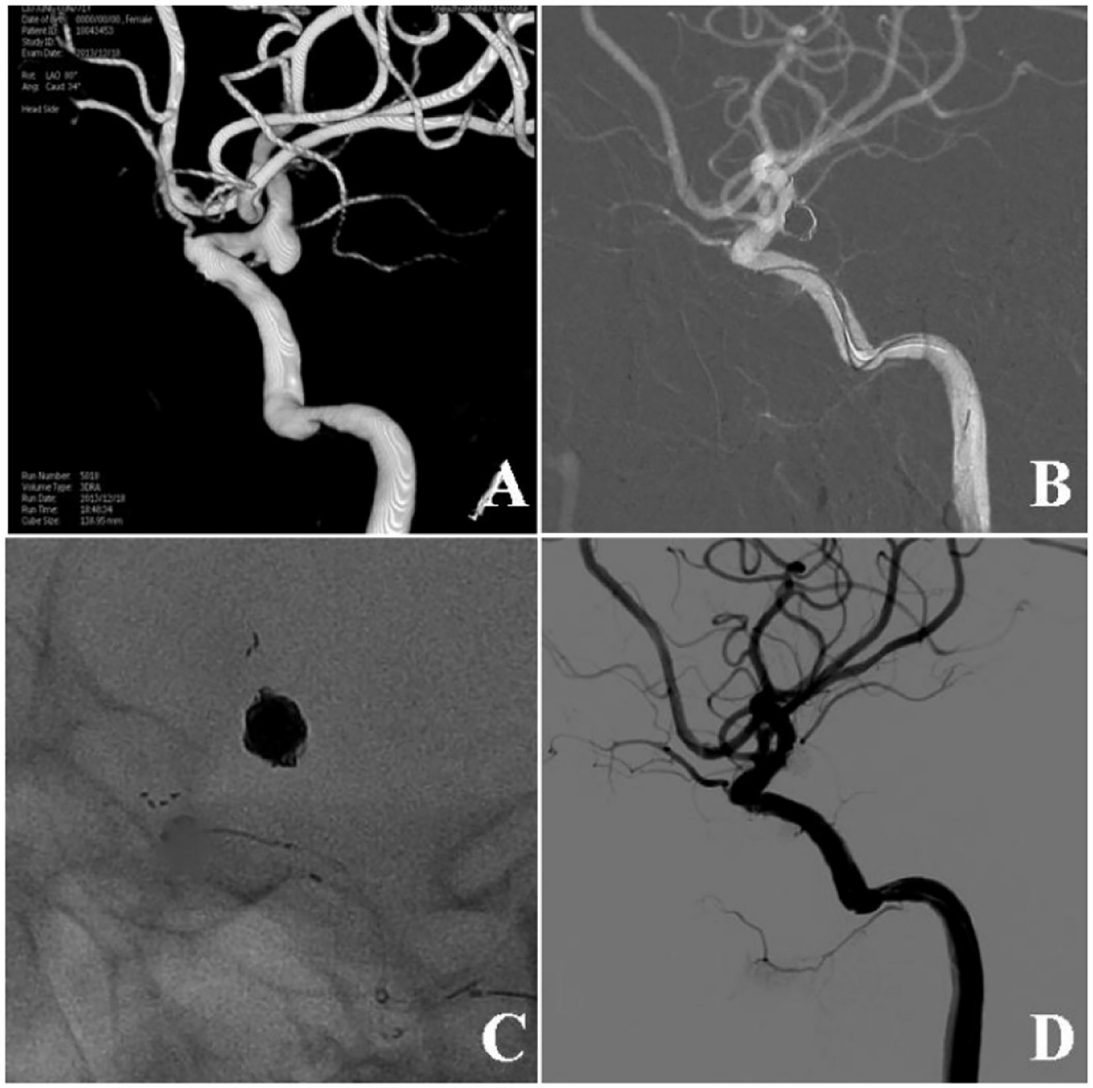
Stent-assisted coiling of a wide-necked ruptured aneurysm at the posterior communicating artery (Pcom). **(A)** Three-dimensional digital subtraction angiography showed a wide-necked aneurysm at the Pcom. **(B)** An Enterprise stent was used for assisting aneurysm coiling. **(C)** The stent and the coil mass are shown. **(D)** Six months following embolization, the aneurysm remained totally occluded.

**Figure 2:**
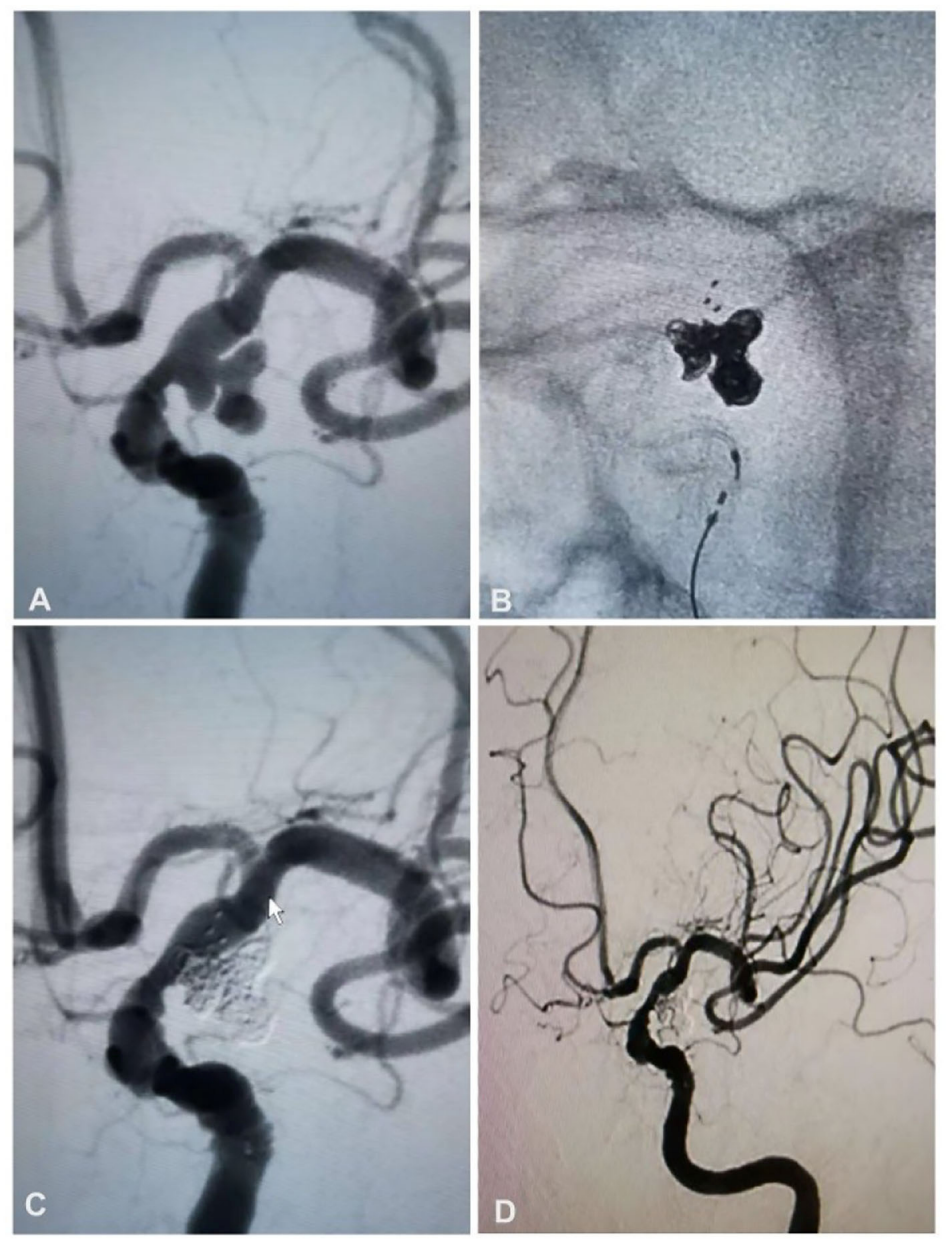
A 63-year-old woman had a ruptured aneurysm at the posterior communicating artery (Pcom) of the left internal carotid artery and was treated with stent-assisted coiling. **(A)** Angiography revealed an aneurysm at the left Pcom. **(B)** Stent-assisted coiling was performed with a Solitaire AB stent (4 × 20 mm) and six coils. **(C)** The aneurysm was totally occluded. **(D)** Follow-up angiography at 6 months revealed that the aneurysm was still totally occluded.

In patients who underwent stent-assisted coiling, two patients experienced deployment of double stents each, but the other patients had only one stent deployed each. The total number of stents deployed was 63 stents, including 36 (57.1%) Solitaire AB stents (Medtronic, Irvine, CA, USA), 22 (34.9%) Enterprise stents (Codman & Shurtleff, Raynham, MA, USA), and 5 (7.9%) Neuroform stents (Stryker, Fremont, CA, USA) (Table 2 and Figures 1, 2). Stent deployment was failed in one patient, with the technical success rate of stenting as 98.4%. The procedure was 169 ± 41 min. Immediate aneurysm occlusion after the treatment was complete in 38 (62.3%) aneurysms, residual neck in 12 (19.7%), and residual aneurysm in 10 (16.4%). In case of failed stent deployment (1.6%) for an aneurysm at the ICA ophthalmic segment, the microcatheter was dislocated and could not be repositioned within the aneurysm sac. Procedure-related complications included in-stent thrombosis in three (4.9%) patients, coil protrusion in three (4.9%), and re-rupture of one (1.6%) aneurysm caused by microcatheter puncture of the aneurysm wall, with a total complication rate of 11.5%. For in-stent thrombosis, 100,000–200,000 units of urokinase were given through a microcatheter for thrombolysis, resulting in complete recanalization 15–30 min later. All complications were managed appropriately without causing any severe sequela.

**Table 2:** Treatment and occlusion degrees of aneurysms.

**Variables**		**Stent-assisted coiling (*n* = 61)**	**Flow diversion (*n* = 53)**	***P***
Treatment modality (*n*)	Stent+coiling	61 (100%)	29 (54.7%)	0.94
	Stent alone	0	24 (45.3%)	
	Double stents	2 (3.3%)	6 (11.3%)	
No. of stents (*n*)		63	59	
Immediate occlusion	Complete occlusion	38 (62.3%)	3 (5.7%)	0.59
	Residual neck	12 (19.7%)	3 (5.7%)	
	Residual aneurysm	10 (16.4%)	47 (88.7%)	
	Failed	1 (1.6%)	0	
Complications	Instent thrombosis	3 (4.9%)	0	0.18
	Coil protrusion	3 (4.9%)	0	
	Rebleeding	1 (1.6%)	1 (1.9%)	
Follow-up	Duration (m, median)	13–49 (29)	14–37 (25)	
	Number	49 (80.3%)	45 (84.9%)	
Follow-up occlusion	Complete occlusion	27 (55.1%)	39 (86.7%)	0.85
	Residual neck	3 (6.1%)	5 (11.1%)	
	Residual aneurysm	5 (10.2%)	1 (2.2%)	
	Recurrence	14 (28.6%)	0	

**Figure 3:**
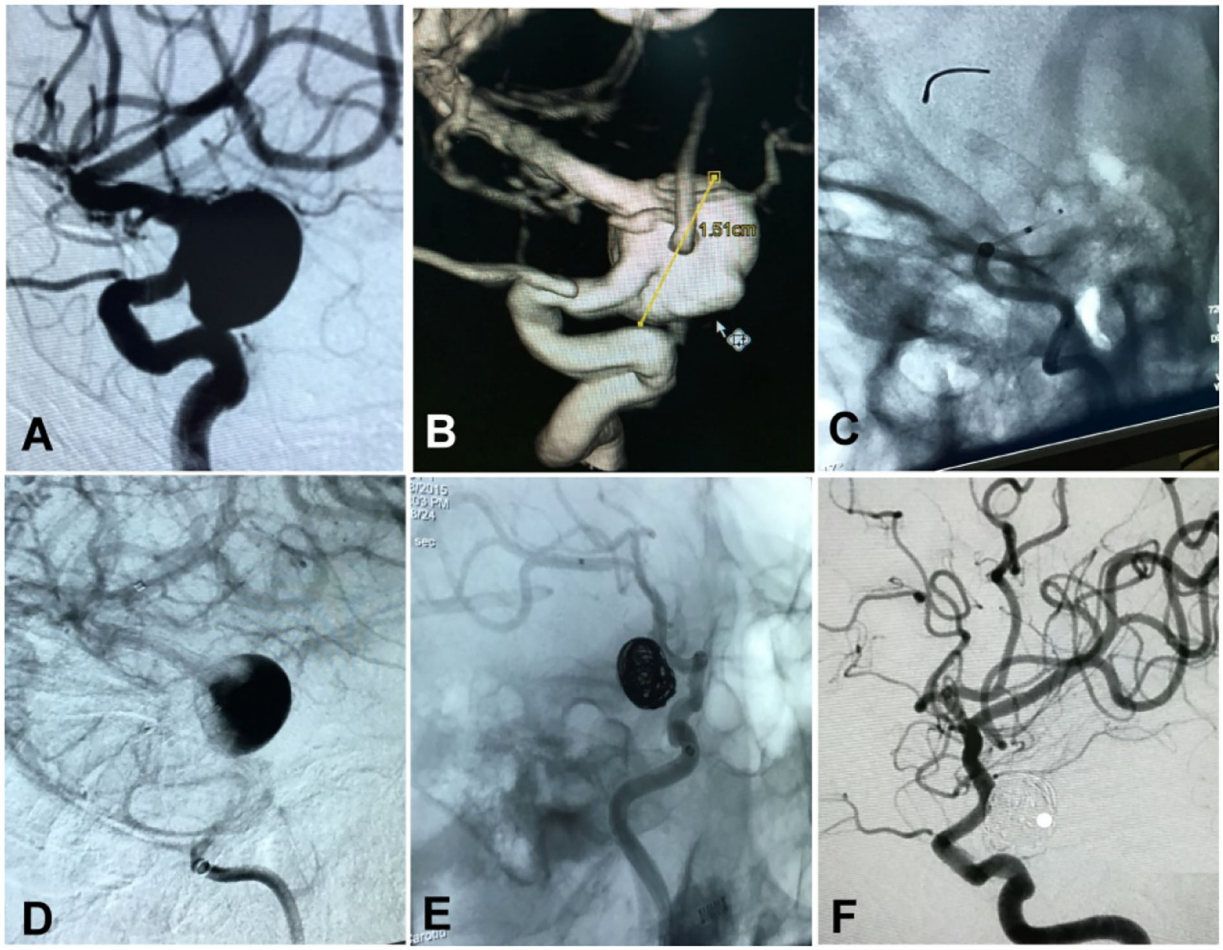
A woman in her 50's had a ruptured aneurysm (Hunt–Hess grade II) measuring 15 × 17 mm at the posterior communicating segment of the internal carotid artery treated with a Pipeline embolization device plus adjunctive coiling. **(A,B)** The aneurysm is shown. **(C)** A Pipeline embolization device of 3.5 × 25 mm was deployed. **(D)** After deployment of the stent, blood flow into the aneurysm cavity was significantly reduced. **(E)** The aneurysm was loosely occluded at the end of embolization. **(F)** At 25-month follow-up, the aneurysm was completely occluded.

**Figure 4:**
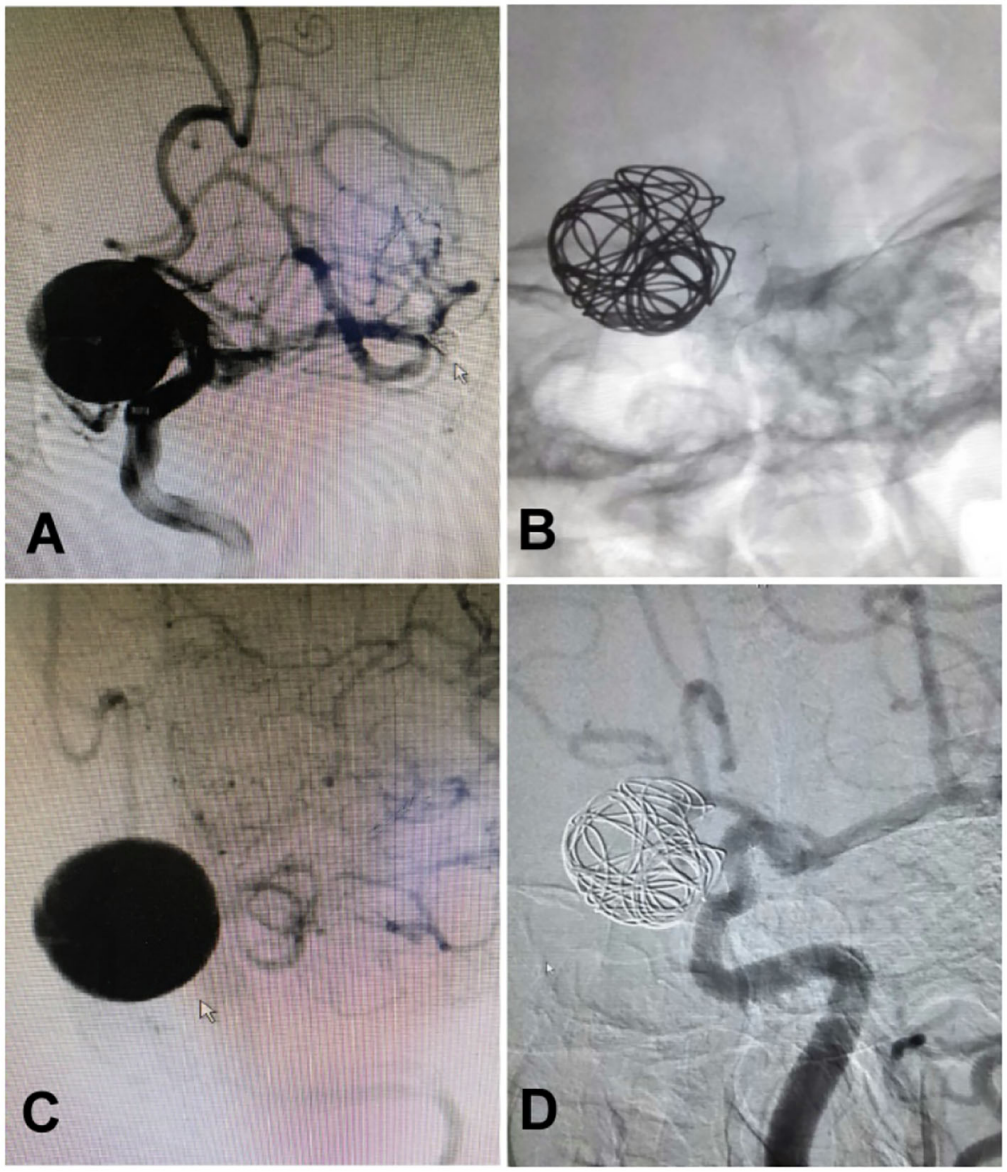
A woman in her 40's with intermittent headache for half a year was found to have an aneurysm measuring 15 × 15 mm at the ophthalmic segment of the internal carotid artery treated with deployment of a Pipeline embolization device and adjunctive coiling. **(A)** The aneurysm was found at the ophthalmic segment. **(B)** A 4.0 × 25 mm Pipeline embolization device was deployed before adjunctive coiling. **(C)** The aneurysm was shown at the end of the embolization. **(D)** One year after embolization, the aneurysm was completely occluded.

In patients experiencing flow diversion treatment, deployment of PED devices alone was performed in each of the 24 (45.3%) patients, flow diversion plus adjunctive coiling in 29 (54.7%), and double PED devices in each of the 6 (11.3%) patients. The total number of PED devices deployed was 59, with the technical success rate of PED deployment of 100% (Table 2 and Figures 3, 4). The procedure time was 122 ± 48 min, which was significantly shorter than that in the stent-assisted coiling group. Immediately after endovascular treatment, complete occlusion was achieved in 3 (5.7%) patients with adjunctive coiling, residual neck in 3 (5.7%), and residual aneurysm in 47 (88.7%). Procedure-related complications included rebleeding of an ophthalmic segment aneurysm in one patient (1.9%) while inserting coils into the aneurysm.

### Follow-up results

Clinical and angiographic follow-up was performed 13–49 months (median 29) after the procedure for 49 (80.3%) patients with stent-assisted coiling. No new neurological symptoms that were related to the stent-assisted coiling procedure were found. Angiographic examination revealed complete occlusion of the aneurysm in 27 (55.1%) aneurysms, residual neck in 3 (6.1%), residual aneurysm in 5 (10.2%), and recurrence in 14 (28.6%), with no symptomatic in-stent stenosis or occlusion. Follow-up was performed 14–37 (median 25) months after the procedure in 45 (84.9%) patients with deployment of flow-diverting devices. No neurological sequela was found in this group. Angiographic imaging demonstrated complete occlusion in 39 (86.7%) patients, residual neck in 5 (11.1%), residual aneurysm in 1 (2.2%), and no aneurysm recurrence. No in-stent stenosis or occlusion was detected. No significant (*p* > 0.05) difference existed in the occlusion status between the two groups.

## Discussion

In this study, investigating the efficacy and safety of stent-assisted coiling in comparison with flow diversion for the treatment of wide-necked intracranial aneurysms, it was found that stent-assisted coiling and flow diversion were both safe and effective for the treatment of wide-necked intracranial aneurysms; however, flow diversion seemed more efficient with more complete occlusion but few recurrence of aneurysms in the long run.

Due to micro invasiveness, few complications, and fast recovery, endovascular embolization has become the first choice of treatment for cerebral aneurysms. Endovascular treatment has been increasingly applied for unruptured wide-necked cerebral aneurysms, with good clinical and angiographic outcomes (2, 5, 6, 11–15). However, ever since the introduction of flow diversion into practice for the treatment of intracranial aneurysms, the use of stent-assisted coiling has been decreasing. Crobeddu et al. have reported a marked decrease from 14.7 to 6.9% (*p* = 0.04) over a 4-year period in the use of stent-assisted coiling in their institute following introduction of the flow diversion technology (16). Flow diversion is a technological advantage compared with the stent-assisted coiling technique because it is a method of reconstruction of the parent artery, encompassing many advantages over stent-assisted coiling such as avoiding coil access to the aneurysm sac with subsequently reduced risk of iatrogenic aneurysm rupture caused by endovascular devices within the aneurysm sac (17). Moreover, adjacent multiple aneurysms can be covered and treated simultaneously in a single procedure with one PED, and the ability to remodel an entire vessel with flow diversion is able to prevent aneurysm recanalization and *de novo* aneurysm formation in the setting of a dysplastic parent vessel (17). In case of large and giant cerebral aneurysms, the technique of stent-assisted coiling may necessitate insertion of a large mass of coils within the aneurysm sac to achieve complete aneurysm occlusion, which may likely aggravate the mass effect-related symptoms caused by the densely packed coils (18, 19). Nonetheless, the flow diverters are able to reconstruct the parent artery lumen and can eliminate the mass effect-related symptoms without inserting coils within the aneurysm sac. Simply deploying a flow diverter at the defect parent artery also means simplification of endovascular embolization operation and decreased radiological irradiation. These advantages have resulted in an increased application of flow diversion but a concurrent decrease in the use of stent-assisted coiling in the treatment of cerebral aneurysms (16, 20).

In one study investigating the effectiveness and safety between the PED and stent-assisted coiling for the treatment of ICA Pcom segment aneurysms (21), including 17 aneurysms treated with the stent-assisted coiling and 21 with PED devices, complete occlusion was achieved in 82.4% of aneurysms in the stent-assisted coiling and 71.4% in the PED devices with no significant (*p* > 0.05) difference at the first angiographic follow-up half a year after the procedure. At the second angiographic follow-up at a median time 8.3 months for the PED group but 27 months for the stent-assisted coiling, complete occlusion was achieved in 70.6% of aneurysms in the stent-assisted coiling but 81% for the PED group. This study (21) confirmed the increased aneurysm recurrence rate but decreased complete aneurysm occlusion rate in patients treated with stent-assisted coiling as well as the increased complete aneurysm occlusion rate but no recurrence in the flow diversion group. In a multicenter cohort study comparing the effect of stent-assisted coiling for 62 aneurysms and PED embolization for 106 aneurysms in the ICA ophthalmic segment (17), the immediate complete occlusion was achieved in 58.1% of aneurysms treated with stent coiling. At the median follow-up of stent-assisted coiling vs. flow diversion (22.5 vs. 8.7 months, *p* = 0.0002), complete occlusion was achieved in 75.9% and 81.1% of aneurysms treated with stent-coiling and PED, respectively, with no significant difference (*p* = 0.516). The need for retreatment was higher with stent coiling. In a study comparing the safety and efficacy of flow diversion and stent-assisted coiling in the treatment of large and giant aneurysms based on a propensity score-matched analysis (22), the complete occlusion rate was significantly higher in the PED cohort than in the conventional stent-coiling cohort at 6-month follow-up. The PED cohort achieved significantly greater improvement but a lower recurrence rate. In our study, the complete occlusion rate of aneurysm immediately after embolization was high in the stent-assisted coiling cohort but lower in the flow diversion group. However, at follow-up of ~2 years, the complete occlusion rate was higher in the flow diversion group but lower in the stent-assisted coiling cohort, which experienced an increased aneurysm recurrence rate.

Our study included aneurysms at different locations like the ICA, anterior and middle cerebral artery, intracranial vertebral artery, and basilar artery, with different sizes of aneurysms treated from small to giant aneurysms. Ruptured and unruptured aneurysms were also involved in our study. Currently, most studies comparing the effect and safety of stent-assisted coiling vs. flow diversion involved only unruptured (23, 24) or ruptured (25) aneurysms, posterior (26) or anterior (27) circulation, small or tiny aneurysms (28). In the procedure-related complications, the stent-assisted coiling involved more complications than those with flow diversion even though there were no significant differences (11.5 vs. 1.9%). The procedure-related complication rate of stent-assisted coiling in comparison with flow diversion had been reported to be of no significant difference (24–28). Chalouhi et al. reported the complication rate in the stent-assisted coiling vs. flow diversion to be 3 vs. 5% (27), including four ischemic events and one rebleeding event in the stent-assisted coiling cohort but one ischemic and one rebleeding event in the PED group, with no procedure-related mortality in either group. Zhang et al. (28) studied 77 small and tiny aneurysms treated with PED deployment in comparison with 281 small and tiny aneurysms treated with stent-assisted coiling but did not find a significant (*p* > 0.05) difference in the complication rate between these two treatment approaches (11.1 vs. 6.1%).

Retreatment is less likely for cerebral aneurysms treated with flow-diverting devices than those treated with the stent-assisted coiling technique because of the high rate of aneurysm occlusion and minimal risk of recurrence achieved with the flow-diverting device (21, 29). Enriquez-Marulanda et al. found no recanalization in the PED group compared with that in the stent-assisted coiling cohort (21). Chalouhi et al. found that a significantly lower rate of retreatment in the PED group than that in the coiling group (5 vs. 32.5%, *p* = 0.003) of patients with small non-complex intracranial aneurysms (30). Xin et al. also found significantly lower rates of retreatment in patients treated with flow diversion than in patients treated with stent-assisted coiling for unruptured cerebral aneurysms (24). In our study, no recurrence was found in 2-year follow-up of aneurysms treated with the flow diverter, consistent with the findings of the above studies.

Studies with three-dimensional models demonstrated that stents deployed at the aneurysm neck can significantly decrease the peak velocity, strengths of vortices and wall shear stress on the inner wall of aneurysms, and that deployment of an additional stent will further decrease these hemodynamic stresses (31, 32). Moreover, experimental and clinical data have demonstrated that the placement of a stent alone across the neck (33–35) of side-wall or fusiform aneurysms could change the intra-aneurysmal hemodynamic status, leading to thrombosis and final obliteration of the aneurysm from blood circulation. Stenting alone provides a novel treatment option for selected cerebral aneurysms, especially the PED flow-diverting device that provides ~30–35% metal surface coverage at nominal expansion–a much higher percent coverage than that provided by conventional intravascular stents (36). The Neuroform stent and the Enterprise stent provide between 6.5 and 9% metal surface coverage when fully deployed in the artery. These properties have enabled the stents to significantly reduce the wall shear stress and flow velocity entering the aneurysm cavity (37). Wang et al. studied the effect of stenting on the wall shear stress and flow velocity into the aneurysm and found that a single PED stent caused less reduction in wall shear stress (51.08%, 0.96 Pa) and velocity (37.87%, 0.0503 m/s), but double PED devices resulted in the most greater reduction in wall shear stress (72.37%, 1.36 Pa) and velocity (54.26%, 0.0721 m/s) (37).

Currently, the PED devices have been refined. The PED Classic device that was the first generation approved in 2011 did not support retrieval after release and had demonstrated some difficulties in deployment as well as poor adherence to arterial wall at tortuous segments of intracranial arteries, which may all increase technique-related procedural complications including arterial dissection and intracranial hemorrhage (38–41). The PED Flex device is the second refined version approved in 2015 to address the disadvantages of the previous-generation device, with improved releasing system, improved resheathing capability, and modified pusher wire (42, 43). Studies have shown improved clinical outcomes of the PED Flex device, with decreases in the surgical time, technical failure, and procedural complications (44–46). With the development of science and technology, flow diverters may be further refined, and the risk profile of flow diverters may be decreased over the years with newer iteration of the devices, resulting in better clinical outcomes. Nonetheless, in the technique of conventional stent-assisted coiling, a conventional stent is still needed to be deployed before inserting coils within the aneurysm sac. No further development in the stent-assisted coiling has been reported in the literature. The stent-assisted coiling technique persists to have a high recurrence rate of aneurysms after embolization because this technique does not significantly decrease the hemodynamic stresses within the parent artery or the aneurysm neck as the flow diverter does. This is probably because the conventional stent does not have a higher metal surface coverage area to reconstruct the parent artery lumen (36), which may constitute the fundamental reason for its higher recurrence rate at follow-up.

Some limitations existed in this study, including a retrospective and single-center study, no randomization, Chinese patients enrolled only, and a small cohort of patients, which may all affect the generalization of the outcomes. Moreover, multiple stents with different brands (Neuroform, Enterprise, and Solitaire) were used in the stent-assisted coiling group, and aneurysms at the posterior and anterior circulation or aneurysms with or without rupture were included in the study, which may also affect the generalization of the study outcome. However, these limitations may better reflect the real clinical setting of endovascular treatment of wide-necked aneurysms using either stent-assisted coiling or flow diversion. Nonetheless, future randomized, multicenter, prospective studies will have to be performed to resolve these issues for better outcomes.

## Conclusion

Stent-assisted coiling may come with more complications but fewer permanent aneurysm occlusions than flow diverters, and flow diverters may be superior to stent-assisted coiling in the treatment of wide-necked intracranial aneurysms, especially in the long-term effect. Nonetheless, further randomized controlled clinical trials are necessary to assess and confirm the advantages and disadvantages of these treatment approaches for wide-necked cerebral aneurysms.

## Data availability statement

The original contributions presented in the study are included in the article/supplementary material, further inquiries can be directed to the corresponding author.

## Ethics statement

The studies involving human participants were reviewed and approved by Ethics Committee of First Hospital of Hebei Medical University. The patients/participants provided their written informed consent to participate in this study.

## Author contributions

C-HL and B-LG: study design and data analysis. HG, J-FL, C-HL, J-WW, and HL: data collection. HL: supervision. All authors: validation. All authors contributed to the article and approved the submitted version.

## Conflict of interest

The authors declare that the research was conducted in the absence of any commercial or financial relationships that could be construed as a potential conflict of interest.

## Publisher's note

All claims expressed in this article are solely those of the authors and do not necessarily represent those of their affiliated organizations, or those of the publisher, the editors and the reviewers. Any product that may be evaluated in this article, or claim that may be made by its manufacturer, is not guaranteed or endorsed by the publisher.
